# Teosinte Inflorescence Phytolith Assemblages Mirror *Zea* Taxonomy

**DOI:** 10.1371/journal.pone.0018349

**Published:** 2011-03-30

**Authors:** John P. Hart, R. G. Matson, Robert G. Thompson, Michael Blake

**Affiliations:** 1 New York State Museum, Albany, New York, United States of America; 2 Department of Anthropology, University of British Columbia, Vancouver, British Columbia, Canada; 3 Department of Anthropology, University of Minnesota, Minneapolis, Minnesota, United States of America; University College London, United Kingdom

## Abstract

Molecular DNA analyses of the New World grass (Poaceae) genus *Zea*, comprising five species, has resolved taxonomic issues including the most likely teosinte progenitor (*Zea mays* ssp. *parviglumis*) of maize (*Zea mays* ssp. *mays*). However, archaeologically, little is known about the use of teosinte by humans both prior to and after the domestication of maize. One potential line of evidence to explore these relationships is opaline phytoliths produced in teosinte fruit cases. Here we use multidimensional scaling and multiple discriminant analyses to determine if rondel phytolith assemblages from teosinte fruitcases reflect teosinte taxonomy. Our results indicate that rondel phytolith assemblages from the various taxa, including subspecies, can be statistically discriminated. This indicates that it will be possible to investigate the archaeological histories of teosinte use pending the recovery of appropriate samples.

## Introduction

Teosinte consists of the undomesticated members of a genus of grasses (*Zea*) native to Mexico and Central America. Doebley and Iltis [1] and Iltis and Doebley [2] provided the current taxonomy of *Zea*, which they divide into two sections (also see [3]). Section Zea includes the annual teosintes *Z mays* ssp. *parviglumis* Iltis and Doebley, ssp. *mexicana* (Schrader) Iltis, and ssp. *huehuetenangensis* (Iltis and Doebley) Doebley. Section Luxuriantes includes the annual teosintes *Z. luxurians* (Durieu and Ascherson) Bird and *Z. nicaraguensis* Iltis and Benz and the two perennial teosintes, *Z. perennis* (Hitchc.) Reeves and Mangelsdorf and *Z. diploperennis* Iltis, Doebley and Guzman.

During the last decade a series of *Zea* genetic studies have elucidated the phylogenetic relationships among the various species and subspecies of this genus (e.g., [3]–[5]). One of the most important outcomes of this research is the demonstration that all varieties of modern maize are genetically more closely related to each other and to *Z. mays* ssp. *parviglumis* than to any other subspecies or species of *Zea*. The phylogenetic proximity between all modern maize varieties and ssp. *parviglumis* supports the hypothesis that this subspecies is the progenitor of maize [4]. Matsuoka et al.'s [4] study examined 99 microsatellites (or SSRs) dispersed throughout the maize genome from 193 different maize plants, 33 ssp. *mexicana* plants, and 34 ssp. *parviglumis* plants, using 4 ssp. *huehuetenangensis* plants as an outgroup. This work indicated that it was most likely ssp. *parviglumis* that was domesticated beginning about 9000 years ago, giving rise to the earliest lineages of teosinte-like maize, which eventually evolved into the remarkable multi-rowed, large naked-kernelled, husk-covered ear of maize that is today one of the world's most important crops.

More recently, Vigouroux et al. [5] extended this analysis by analyzing similar microsatellites from 771 additional maize and 5 ssp. *parviglumis* plants (as their outgroup). The combined data from these studies resulted in the broadest geographic coverage so far available, encompassing nearly all of the known races and varieties of maize from Canada to Chile. Vigouroux et al. found that allelic variation at 96 of the original 99 microsatellites allowed them to place all of the maize plants into four main clusters. These clusters (Highland Mexican, Tropical Lowland, Andean, and Northern US) correspond to the chronological spread of maize from highland Mexico northwards into the southwestern US (and then subsequently into northeastern and eastern US and southeastern Canada) and southwards into the tropical lowlands above the equator (and then south into the Andean highlands and from there to the southern lowlands of South America).

**Figure 1:**
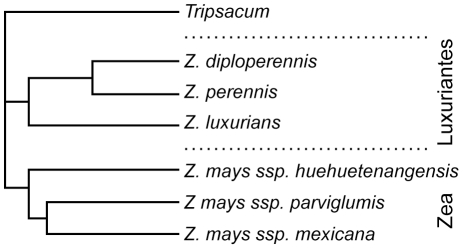
Simplified tree showing the likely genetic relationships among the teosintes when using *Tripsacum* as the outgroup. (Based on [3], Figure 3).

While the Matusuoka et al. [4] and the Vigouroux et al. [5] phylogenies provide a plausible hypothesis on how maize evolved from an ancestral population of *Z*. *mays* ssp. *parviglumis* on the Pacific slope of west central Mexico, they did not elucidate the genetic relationships among the various species and subspecies of teosinte. Using a sample of 237 teosinte plants from all species and subspecies, and encompassing the geographic range from northwestern Mexico to Nicaragua, as well as two individual *Tripsacum* plants as their outgroup (one each of *T. zopilotense* and *T. peruvianum*), Fukunaga et al. [3] analyzed the allelic diversity in the same (or similar) set of microsatellites (SSRs) as used in the two maize studies. Their results confirm that across a broad set of SSRs, the teosintes can be divided into the two sections previously suggested by Doebley and Iltis [1]. They suggest that *Z. luxurians* is either ancestral to section Zea, or, more likely, the root lies somewhere between (i.e., ancestral to both) sections Zea and Luxuriantes (Figure 1).

These three studies suggest that the various teosinte species and subspecies should have predictable genetic and phenotypic similarities based on their ancestral proximity to one another. Dorweiler and Doebley [6] and Wang et al. [7] have demonstrated that the gene responsible for the development of glumes in *Zea*, teosinte glume architecture1, *tga1*, also controls the deposition of silica that produces opaline phytoliths in the cells of glumes. One of the most significant events in the domestication process was the change in the expression of *tga1*, enabling the sealed, indurated fruitcase of teosinte to open up, allow a naked grain, and form a less lignified, softer glume. Work on phytolith assemblages from different taxa of *Zea* has been progressing for many years. Although studies have focused on differentiating between the phytoliths produced in the glumes of maize and teosinte based on proportions of phytolith types (e.g., [8]–[10]), to date there has been no determination as to whether the glume (rondel) phytoliths from the various teosinte species and subspecies can be discriminated. The ability to make these identifications will be critical to elucidating human use of teosinte both prior to and following the evolution of maize. If it is possible to differentiate between the rondel phytoliths produced by the various teosinte taxa, reconstructing the histories of human–teosinte interactions both before and after the evolution of maize will be enhanced. Even though teosinte is assumed to have been an important resource as it evolved into maize, currently it is almost invisible in the archaeological record. Here we test two hypotheses:

H_0_: Teosinte rondel phytolith “profiles” do not reflect teosinte phylogeny.

H_1_: Teosinte rondel phytolith "profiles" reflect teosinte phylogeny.

We show that it is possible to discriminate between rondel phytolith assemblages of the various taxa using the proportions of as few as two morphological categories. The implication is that teosinte rondel phytolith assemblages are highly reflective of *Zea* phylogeny and that H_0_ can be rejected. This demonstrates that the ability to differentiate maize from teosinte is not the only taxonomic utility of *Zea* phytolith assemblages.

## Results

**Figure 2:**
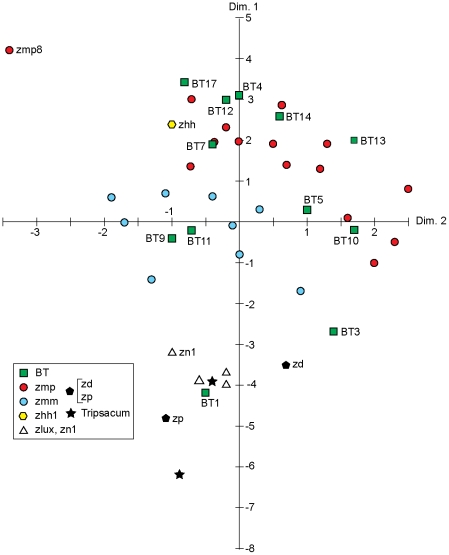
Metric Multidimensional Scaling (MDS) of unstandardized Euclidean distances based on 45 rondel classes, dimensions 1 and 2. Dimension 1 accounts for 65% of trace, dimension 2, 14%. Samples coded for teosinte taxa, with BT = Blind Test, Tripsacum = Tripsacum.

**Figure 3:**
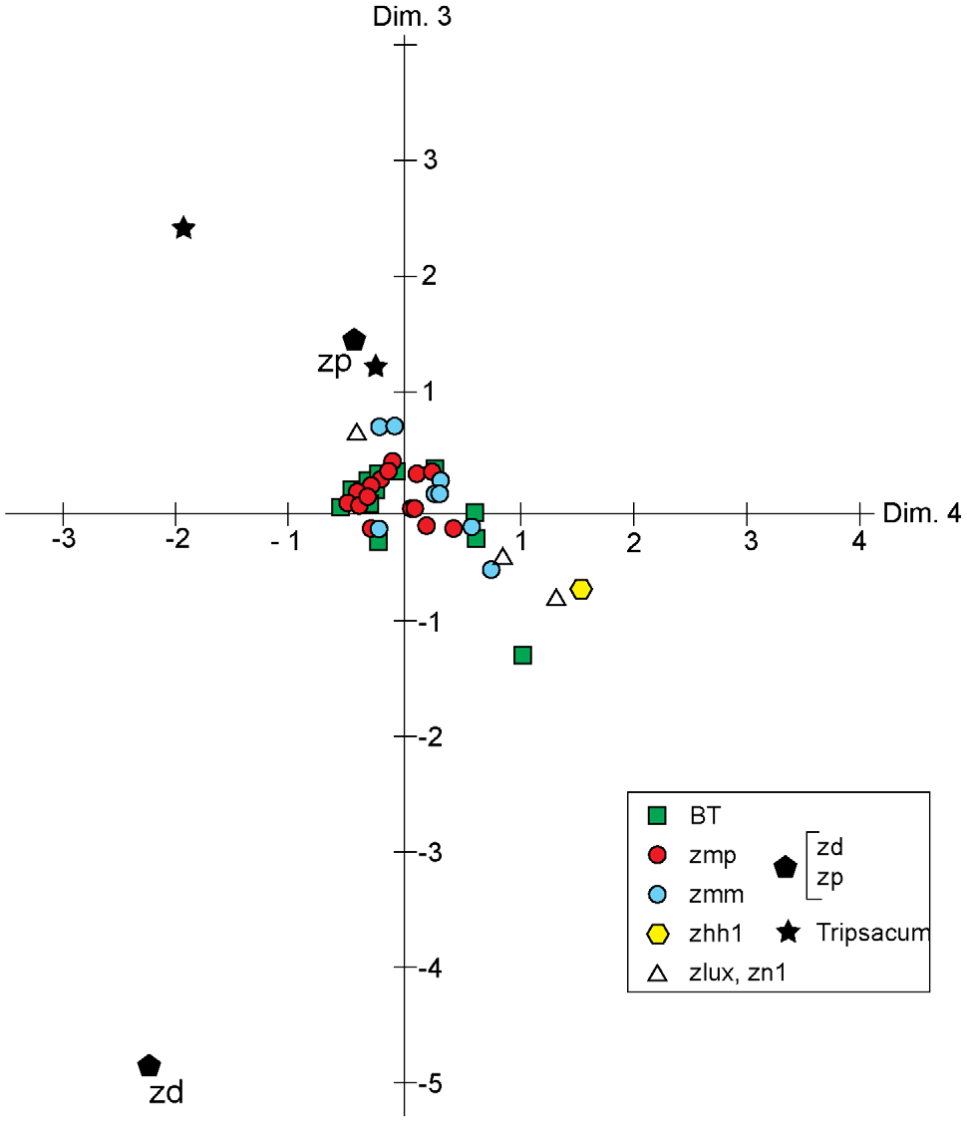
Metric Multidimensional scaling, Dimensions 3 and 4. Coding as in Figure 2. Dimension 3 accounts for 9.1% of trace, dimension 4, 4.5% of trace.

Metric Multidimensional Scaling (MDS) [11], [12] using unstandardized Euclidean distances produced clear results (Figure 2) with plots identical (except for the scale) to those produced by Principle Coordinates Analysis [13]–[15] (not shown). MDS groups together known teosinte taxa assemblages, including those of *Z. mays* ssp. *mexicana* and *Z. mays* ssp. *parviglumis,* largely in accord with *Zea* taxonomy (Figures 2 and 3). A very similar pattern for the first two dimensions was found using chord distances (not shown) except that *Z. mays* ssp. *mexicana*–*parviglumis* separation was not perfect. However, the first four dimensions of the chord distance results could be rotated slightly, to achieve a perfect separation of these subspecies.

The third and fourth dimensions of the MDS results using Euclidean distances are shown in Figure 3, with most known teosinte samples grouped with other samples with the same taxon. These two dimensions account for only 13.62 percent of the total squared distance from the centroid (variance). No other dimension accounted for more than 4 percent of the variance. These results clearly demonstrate that variation in rondel morphological category abundance between teosinte taxa (and *Tripsacum*) assemblages corresponds very closely to the Fukunaga et al. [3] taxonomy.

**Figure 4:**
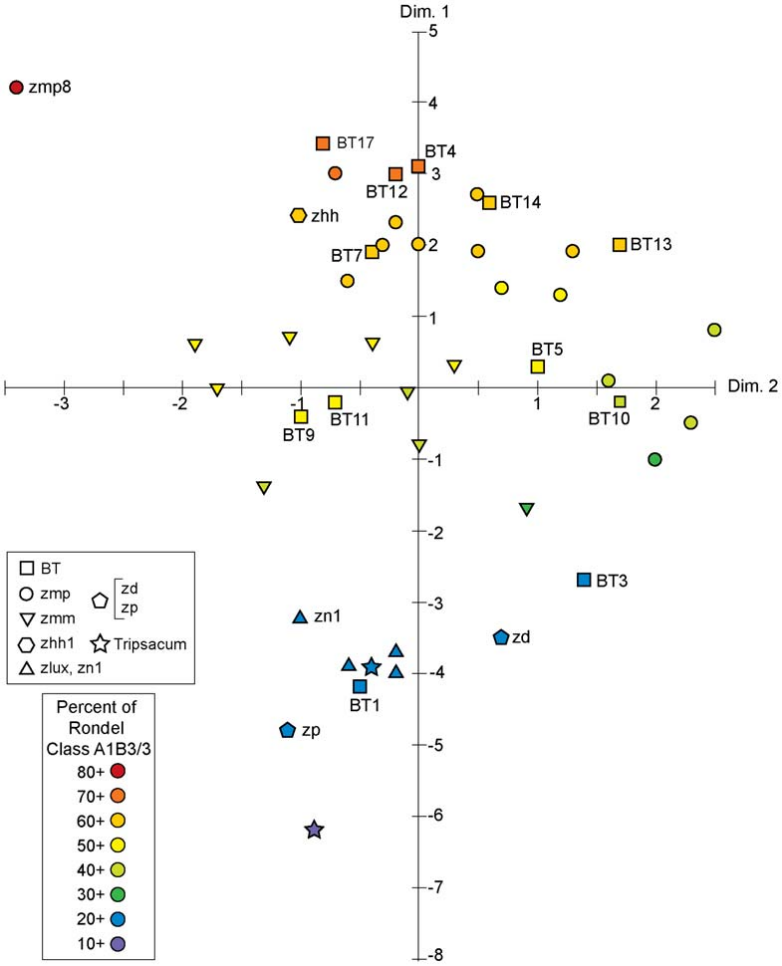
MDS results of Figure 2, coded for abundance of rondel class A1B3/3. Samples coded for teosinte taxa.

**Figure 5:**
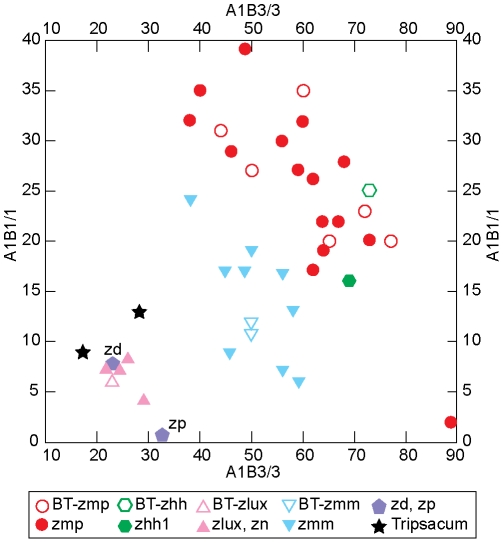
Teosinte samples plotted against abundance of rondel classes A1B3/3 and A1B1/1. Samples coded as in Figure 4.

There is a high Spearman rank correlation of +0.977 between MDS dimension 1 and the abundance of morphological class A-1-B-3/3, the most common rondel category. Dimension 2 has a linear correlation of +0.8598 with category A-2-B-3/4, the second most abundant rondel form and dimension 3 has a linear correlation of –0.5520 with A-1-B-1/1. Two other correlations between dimensions and morphological categories were also noted, +0.6841 of A-2-B-1/4 with dimension 2 and +0.4855 of C-1-D-1-1/1 with dimension 3. Figure 4 shows the first two dimensions of the MDS with the intensity of the color indicating the abundance of A-1-B-3/3, a visual representation of its relationship with the first dimension. It is apparent, then, that three relatively abundant rondel categories can be used to distinguish between the various teosinte taxa. In fact, plotting the samples according to the abundance of two of the most highly correlated rondel categories, A-1-B-3/3 and A-1-B-1/1, results in a separation into the appropriate taxa (Figure 5). Eleven blind test (BT) phytolith assemblages were assigned to the class of their nearest neighbor in the Euclidean distance matrix using the full set of 45 morphological categories present in teosinte samples (Table 1). The assignments are in agreement with known taxa in all but one case for a 91% correct classification.

**Table 1:** Nearest neighbor assignment of blind test (BT) phytolith assemblages.

No.^1^	Classification	Nearest	Distance	correct?
		neighbor		
BT1	*Z. luxurians/nicaraguensis* (P1306615)	zmlx2	1.98	Yes
BT4	*Z. mays* ssp. *huehuetenangensis* (Ames 21889)	zmp10	1.55	No**
BT5	*Z. mays* ssp. *parviglumis* (Ames 21785)	zmp12	1.13	Yes
BT7	*Z. mays* ssp. *parviglumis* (PI 331785)	zmp14	1.18	Yes
BT9	*Z. mays* ssp. *mexicana* (Ames 8083)	zmm7	1.43	Yes
BT10	*Z. mays* ssp. *parviglumis* (PI 566689)	zmp11	1.14	Yes
BT11	*Z. mays* ssp. *mexicana* (PI 566674)	zmm7	1.17	Yes
BT12	*Z. mays* ssp. *parviglumis* (Ames 21890)	zmp5	1.24	Yes^2^
		zhh1		
BT13	*Z. mays* ssp. *parviglumis* (PI 566688)	zmp12	1.16	Yes
BT14	*Z. mays* ssp. *parviglumis* (PI 212889)	zmp6	1.10	Yes
BT17	*Z. mays* ssp. *parviglumis* (PI 384066)	zmp5	1.38	Yes

1 BT3 was excluded because it had apparently been misidentified in its original repository prior to the present study.

2 Iltis and Doebley [2] classify *huehuetenangensis* as a variety of *parviglumis*.

Using multiple discriminant analysis (MDA), the three rondel categories most highly correlated with MDS dimensions 1–3 resulted in a perfect classification of the known assemblages (*Z. mays* ssp. *parviglumis*, *Z. mays* ssp. *mexicana*, Section Luxuriantes) and in the assignment of the BTs to taxa (not shown), excluding BT4 which did not fit into the categories (thereby reducing our sample of BTs from eleven to ten). The results of the MDA indicate that rondel category A-2-B-3/4 does not contribute significantly to the function. Eliminating that category resulted in a two-variable function that assigned all known and BT samples to their correct categories (Tables 2 and 3).

**Table 2:** Two-variable (A-1-B-3/3, A-1-B-1/1) MDA classification matrix of known assemblages (direct method).

Observed	Predicted			
	Lux	Zmm	Zmp	Percent
Section Luxuriantes (Lux)	6	0	0	100
*Z. mays* ssp. *mexicana* (Zmm)	0	9	0	100
*Z. mays* ssp. *parviglumis* (Zmp)	0	0	15	100
Total	6	9	15	100

**Table 3:** Two-variable (A-1-B-3/3, A-1-B-1/1) MDA classification matrix of BT assemblages (direct method).

Case	Actual	Predicted^1^		
		1	2	3
BT1	*Z. mays luxurians/Z. nicaraguensis* (P1306615)	Lux	Zmm	Zmp
BT5	*Z. mays* ssp. *parviglumis* (Ames 21785)	Zmp	Zmm	Lux
BT7	*Z. mays* ssp. *parviglumis* (PI 331785)	Zmp	Zmm	Lux
BT9	*Z. mays* ssp. *mexicana* (Ames 8083)	Zmm	Zmp	Lux
BT10	*Z. mays* ssp. *parviglumis* (PI 566689)	Zmp	Zmm	Lux
BT11	*Z. mays* ssp. *mexicana* (PI 566674)	Zmm	Zmp	Lux
BT12	*Z. mays* ssp. *parviglumis* (Ames 21890)	Zmp	Zmm	Lux
BT13	*Z. mays* ssp. *parviglumis* (PI 566688)	Zmp	Zmm	Lux
BT14	*Z. mays* ssp. *parviglumis* (PI 212889)	Zmp	Zmm	Lux
BT17	*Z. mays* ssp. *parviglumis* (PI 384066)	Zmp	Zmm	Lux

1 1 = highest predicted group, 2 = second highest predicted group, etc.

The correct assignment of BT samples to their known biological taxa supports the earlier results, confirming that the abundance of rondel categories matches very closely teosinte phylogeny based on microsatellite analysis [3]. Our collective results indicate, then, that inflorescence rondel phytoliths can be used to accurately discriminate between the various teosintes.

In an on-going investigation of Mexican maize phytolith assemblages, an MDA using only three rondel categories (A-2-B-3/3, A-1-B-4/4, and C-4-D-4-4/4) shows near perfect classification of maize and teosinte (Table 4). These results further demonstrate the ability of *Zea* taxa to be distinguished based on rondel phytolith assemblages using only a very few morphological categories.

**Table 4:** Three-variable (A-2-B-3/3, A-1-B-4/4, C-4-D-4-4/4) MDA classification matrix of teosinte and Mexican maize samples (direct method).

Phytolith Assemblages	Predicted Group Membership		
	Teosinte	Maize	Total
Teosinte Samples	30	0	30
Maize Samples	1	28	29
"Unknown" Samples (Teosinte)^1^	14	1	15

1 MDA is sensitive to unequal sample sizes, so 30 of the teosinte samples were used as "Knowns" and the remaining 15 were used as "unknowns", a recommended procedure for cases like this [16 p51].

## Discussion

Our results clearly show that H_0_ can be rejected while H_1_ cannot; rondel phytolith profiles closely reflect current *Zea* phylogeny based on microsatellite DNA. The results of both scaling and discriminant analyses led to the identification of the same rondel morphological categories as being the important discriminators. Plots using the frequencies of only two rondel categories result in the same pattern without multivariate manipulation. Our analysis demonstrates that there are consistent proportions of these rondel categories within each teosinte taxon and that there are consistent differences in proportions between the various taxa. Expanding on an idea originally suggested by Piperno and Pearsall [10], we hope that our analysis will eventually help in identifying specific changes in inflorescence phytolith assemblages during the long course of maize's evolution. This in turn will help elucidate human-teosinte interactions both before and after the evolution of maize. For example, one hypothesis for the relative absence of teosinte in the archaeological record is that teosinte (and early maize) was used primarily as a source of sugar rather than as grains [17], [18]. Another hypothesis is that teosinte and very early maize inflorescences were consumed as raw greens while still immature (Pearsall cited in [19]). The most likely source of rondel phytolith assemblages for analysis are quids and coprolites recovered from dry caves in central Mexico such as the Tehuacan Valley caves [20], [21], Guilá Naquitz cave in Oaxaca [22], and the Tamaulipas caves [23].

## Materials and Methods

Fruitcases from teosinte of known genetic background were analyzed, and a database reflecting their phytolith assemblage phenotypes was developed. Germplasm from each of Z*ea* taxa is archived at the North Central Plant Introduction Station (NCPI). These samples are of known genetic background, and the populations from which the kernels were collected are known. This is the source from which the plants used in this study were grown, augmented by samples obtained from Mary Eubanks. Teosinte fruitcases were recovered from known samples by randomly removing three fruitcases from each plant. The samples obtained from NCPI totaled from 25 to 100 seeds (the amount of seed in each sample was determined based on availability by NCPI). Samples obtained from Mary Eubanks contained five seeds.

The harvested fruitcases were treated with heated nitric acid, which dissolved the organic matter, leaving the opal phytoliths. Following nitric acid removal of organics, the solutions were placed in centrifuge tubes and centrifuged at 3000 rpms for 15 minutes at a time to concentrate the phytoliths in the bottom of the test tubes. The supernatant nitric acid was then pipetted off and replaced with distilled water. After five repetitions of centrifuging, pipetting off the supernatant liquid, and replacing with distilled water, the procedure was duplicated, replacing the distilled water with ethanol.

Phytoliths were then pipetted onto slides and after the alcohol evaporated the phytoliths were sealed under a cover slip with permount. Each of 100 rondel phytoliths from every sample was assigned to a morphological category using a morphological taxonomy originally developed by Mulholland and Rapp [24] for Poaceae and subsequently expanded and modified by Thompson based on his experience with *Zea* [25].

**Figure 6:**
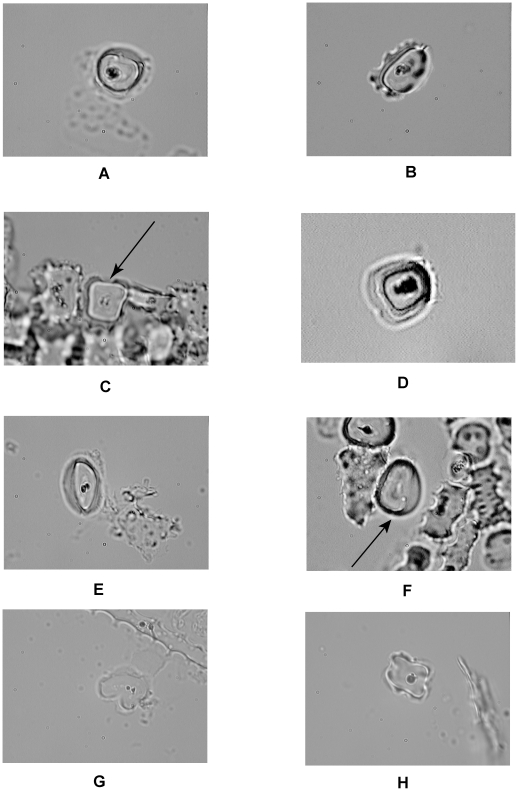
Photographs of representative of the important discriminating rondel phytolith categories listed in Table 5. Original magnification 1000× except D at 400×. Arrows in C and F indicate the phytolith belonging to the relevant category.

Each rondel phytolith was examined in planar (upright) view for coding. The taxonomy produces an alpha-numeric code for each rondel phytolith based on the shapes of the thin (larger) and thick (smaller) faces. For example, code A-1-B-1/1 represents a rondel phytolith with a thin face that is a complete circle without decorations and is approximately the same size as the circular thick face that has decorations [25], [26]. Listings of attributes for various rondel phytolith categories identified above as important discriminators between the *Zea* taxa are presented in Table 5. Images of representative phytoliths in these categories are shown in Figure 6.

**Table 5:** Descriptions of important discriminating rondel phytolith categories mentioned in text.

Code	Phytolith Category Attributes and Corresponding Figure Number
A-1-B-1/1	A–thin face is a complete circle or oval outline, without decorations.
	1–thin face is approximately the same size as the thick face.
	B–thick face is a complete circular or oval outline, with decorations present
	1/1–both faces are circular
	Figure 6a
A-1-B-3/3	A–thin face is an complete circle or oval outline, without decorations
	1–thin face is approximately the same size as the thick face
	B–thick face is a complete circular or oval outline, with decorations present
	3/3–both faces are oval
	Figure 6b
A-1-B-4/4	A–thin face is an complete circle or oval outline, without decorations
	1–thin face is approximately the same size as the thick face
	B–thick face is a complete circular or oval outline, with decorations present
	4/4–both faces are oval with squared corners on the ends
	Figure 6c
A-2-B-1/4	A–thin face is an complete circle or oval outline, without decorations2– thin face is substantially different in size from the thick faceB–thick face is a complete circular or oval outline, with decorations present1–thin face is circular4– thick face is oval with squared corners on endsFigure 6d
A-2-B-3/3	A–thin face is an complete circle or oval outline, without decorations.
	2–thin face is substantially different in size from the thick face
	B–thick face is a complete circular or oval outline, with decorations present
	3/3–both faces are oval
	Figure 6e
A-2-B-3/4	A–thin face is an complete circle or oval outline, without decorations.
	2–thin face is substantially different in size from the thick face
	B–thick face is a complete circular or oval outline, with decorations present
	3–thin face is oval
	4–thick face is oval with squared corners on ends
	Figure 6f
C-1-D-1-1/1	C–1- thin face has one indentation
	D–1- thick face has one indentation
	1/1–both faces are circular
	Figure 6g
C-4-D-4-4/4	C-4–thin face has three indentations
	D-4–thick face has four indentations
	4/4–both faces are oval with squared corners on the ends
	Figure 6h

Following [25], [27]–[29] we used a quantitative approach by measuring similarity on the basis of the abundance of the rondel morphological categories. Previous studies have demonstrated that rondel phytolith assemblages can be used to distinguish maize from non-maize grasses from both modern and archaeological samples. Hart and Matson [29] were able replicate the results in Hart et al. [28] with multivariate discriminant analysis (MDA) using a substantially reduced number of rondel morphological categories. The present sample consists of rondel assemblages from 43 teosinte and two Tripsacum plants, with each having from 99 to 101 rondels (mode = 100) assigned to 45 morphological categories (see supplemental data). Unlike much previous work using morphological rondel categories (e.g., [27]) no size information is used in the present analysis.

Previous analyses of rondel assemblages used squared chord distances [30], [31] as a measure of similarity. In the current analyses we found that unstandardized Euclidean distances [11], [32] result in clearer patterns with the teosinte dataset. We analyzed the resulting 45×45 distance matrix with a number of statistical techniques, including metric Multidimensional Scaling (MDS) [12].

Blind tests were conducted to confirm the ability of rondel phytolith assemblages to reflect the taxa from which they were recovered. Blind test samples were prepared by Mary Eubanks. These tests were doubly blind in that the analyst classifying the sample did not know to which taxa the sample belonged and the analyzers of the data initially had no knowledge of the meaning of “BT” in the dataset. We chose to initially use a procedure to classify the blind test (BT) phytolith assemblages similar to that developed by Hart et al. [28] to assign archaeologically derived phytolith assemblages to either maize or indigenous grass categories.

Following Hart and Matson [29] we subsequently used multivariate discriminant analysis (MDA) as a further test of the hypotheses. For this analysis we assigned all of the known phytolith assemblages to one of three categories: *Zea mays* ssp. *mexicana*, *Z. mays* ssp. *parviglumis*, and section Luxuriantes including *Z. luxurians*, *Z. diploperennis*, *Z. perennis*, and *Z. nicaraguensis* [3].
